# Development of a 3-PRR Precision Tracking System with Full Closed-Loop Measurement and Control

**DOI:** 10.3390/s19081756

**Published:** 2019-04-12

**Authors:** Ling-bo Xie, Zhi-cheng Qiu, Xian-min Zhang

**Affiliations:** Guangdong Provincial Key Laboratory of Precision Equipment and Manufacturing Technology, School of Mechanical and Automotive Engineering, South China University of Technology, Guangzhou 510640, China; lingboxie@163.com (L.-b.X.); zhchqiu@scut.edu.cn (Z.-c.Q.)

**Keywords:** full closed-loop, laser displacement sensor, linear grating encoder, 3-PRR parallel platform, precision tracking

## Abstract

A 3-PRR (three links with each link consisting of a prismatic pair and two rotating pairs) parallel platform was designed for application in a vacuum environment. To meet the requirement of high tracking accuracy of the 3-PRR parallel platform, a full closed-loop control precision tracking system with laser displacement sensors and linear grating encoders was analysed and implemented. Equally-spaced laser displacement sensors and linear grating encoders were adopted not only for measurement but also for feedback control. A feed-forward control method was applied for comparison before conducting the closed-loop feedback control experiments. The closed-loop control experiments were conducted by adopting the PI (proportion and integration) feedback control and RBF (radial basis function) neural network control algorithms. The experimental results demonstrate that the feed-forward control, PI feedback control, and RBF neural-network control algorithms all have a better control effect than that of semi-closed-loop control, which proves the validity of the designed full closed-loop control system based on the combination of laser displacement sensors and linear grating encoders.

## 1. Introduction

Precision positioning systems increasingly require a high positioning accuracy and a large travel range [1,2], and play an important role in the fields of planar manipulations, industrial robots, measurement systems, and so on [3,4,5]. To meet the requirements of high positioning accuracy and long travel range, parallel robots have been developed and designed as a suitable alternative to serial robots [6]. Many types of parallel mechanisms have been developed in recent years, such as 3-RRR (three degrees of freedom with each branch consisting of three rotating pairs) and 3-PRR (three links with each link consisting of a prismatic pair and two rotating pairs) planar parallel mechanisms [7]. The 3-PRR planar parallel mechanism is investigated in this paper. The input motions of the different branches of 3-PRR are coupled to each other and interference phenomena are inevitable, which results in tracking errors during tracking of the desired trajectory. Therefore, to improve the tracking accuracy, it is important to precisely identify certain parameters of the parallel mechanism. In practice, calibration is an effective method to improve the positioning accuracy of the parallel mechanism [8]. A calibration method combining the error model and assistant measurement was proposed, and experimental results demonstrated that this method can achieve better calibration for a 3-PRR parallel platform [9].

After calibration, because the semi-closed-loop control system is susceptible to external interference, friction, and wear, the absolute positioning accuracy will gradually become worse. An observer can be designed to estimate the end-effector state information without the external sensor [10]. However, the noise and errors introduced from the observation seriously worsen the positioning accuracy. Therefore, an external sensor is needed to measure the motion of the parallel mechanism for closed-loop feedback control, and a full closed-loop control method can be an ideal choice [11,12,13]. For many parallel multi-degrees-of-freedom mechanisms, visual inspection is the most widely used method [14,15]. Fatikow et al. [16] used visual feedback to extend automated closed-loop positioning based on different external sensors, and the integration of mobile micro-robots into a specific system can be done fully automatically. A micro-vision system (MVS) was used to obtain feedback signals online, which illustrates that a full closed-loop tracking control can be developed to enhance the positioning accuracy of micro/nano-positioning systems [17]. The main disadvantage of visual inspection is that the sampling rate is too slow to measure a relatively rapid tracking motion. In practice, other sensors can be used to obtain feedback for the parallel mechanism, such as capacitance sensors, eddy current sensors, resistance strain gauges, and laser displacement sensors. Three capacitance sensors were installed at the end of a planar three-degrees-of-freedom (3-DOF) nano-positioning platform to realize feedback control, and the trajectory tracking accuracy of the mechanism was improved by using the traditional PID (proportion, integration, and differentiation) control algorithm [18]. Kuan et al. [19] built a full closed-loop control system for a three-DOF micro motion stage using eddy current sensors, and closed-loop positioning control was achieved using a standard PI controller. A laser displacement sensor was used to form a full closed-loop control system in a 6-DOF precision positioning platform, and the experimental results showed good measurement performance [20]. All capacitance sensors, eddy current sensors, and resistance strain gauges have very small measurement ranges, which are not suitable for large travel measurements. Because laser displacement sensors have the advantages of high sampling rate, high measurement accuracy, and a relatively large measurement range, they can be used for the measurement of the fast tracking motion of a precision tracking platform. Therefore, the laser displacement sensor was selected for the full closed-loop measurement and control of 3-PRR parallel mechanism in this paper.

Conventional PID feedback control can be used for trajectory tracking with asymptotic stability if the control parameters are carefully selected [21]. However, if the control gains are set at random, then the system will enter an infinite loop or lose control, as shown in later experiments. Therefore, it is necessary to make the PID parameters scientifically and effectively self-adjust. Ouyang et al. [22] improved the trajectory tracking performance of a 2-DOF parallel mechanism by using an adaptive switching learning PD control method, and the convergence rate was faster than that of the adaptive iterative learning control method proposed by others in the literature. In addition, many researchers have used different kinds of neural networks to adjust the PID parameters and acquired good control performance [23,24]. In view of its strong learning ability and fast convergence ability, the RBF (radial basis function) [25,26] neural network was selected to improve the tracking accuracy of the 3-PRR parallel mechanism in this paper.

The above analysis shows that few people are designing and investigating a full closed-loop control 3-PRR precision tracking system with laser displacement sensors and neural-network-based control algorithms. Compared with visual inspection and other sensors, the laser displacement sensor has certain advantages, such as high sampling frequency and high measurement accuracy, which will be demonstrated in later experiments. In view of its strong learning ability and fast convergence ability, the RBF neural network control algorithm has been designed to improve the tracking accuracy for different tracking trajectories. The rest of this article is organized as follows: Section 2 introduces the experimental setup, which is divided into three parts, the experimental setup, deviation of installing angle and equally-spaced sensors, and the measurement evaluation. A feed-forward control experiment is presented in Section 3 that shows that feed-forward control can reduce tracking errors to an extent. A PI feedback control experiment is presented in Section 4 that shows that when the PI parameters are carefully selected to avoid entering an infinite loop or losing control, the PI feedback control algorithm can achieve a relatively good control effect. Section 5 presents the RBF neural-network-based control algorithm, and the experimental results illustrate that the neural-network-based control algorithm can dramatically improve the tracking accuracy. Conclusions are drawn in Section 6.

The contribution of this paper is mainly two-fold. First, a three-degrees-of-freedom full closed-loop control precision tracking system was developed and analysed in the paper. The precision tracking system with three equally-spaced laser displacement sensors and linear grating encoders for full closed-loop control is illustrated in detail, and demonstrates high measurement accuracy. In fact, the six-degrees-of-freedom motion of more complicated mechanisms can be measured by more equally-spaced laser displacement sensors in combination. Second, experimental studies of semi-closed-loop control, feed-forward control, PI feedback control, and RBF neural-network control methods were carried out. The experimental results demonstrate that all of the feed-forward control, PI feedback control, and RBF neural network control algorithms have better control effects than that of semi-closed-loop control, which proves the validity of the designed full closed-loop control system based on the combination of laser displacement sensors and linear grating encoders.

## 2. Introduction of the Experimental Setup

### 2.1. Experimental Setup

**Figure 1:**
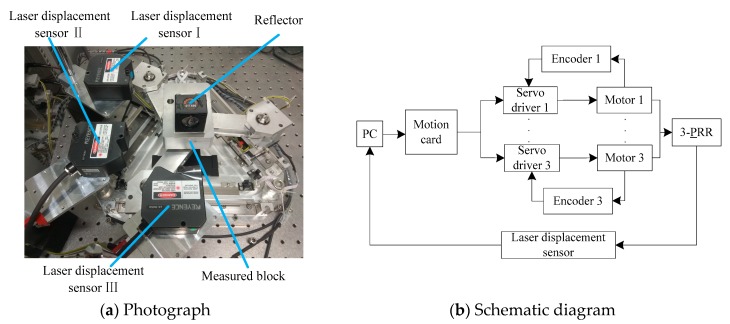
Photograph and schematic diagram of the closed-loop feedback control of the precision tracking system.

**Figure 2:**
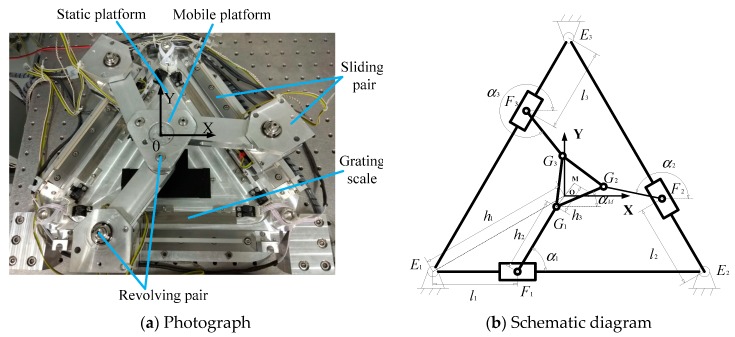
Photograph and schematic diagram of the 3-PRR (three links with each link consisting of a prismatic pair and two rotating pairs) parallel platform.

As shown in Figure 1, the experimental setup consists of three equally-spaced laser displacement sensors, three linear grating encoders, a computer, a motion card, three servo drivers, and motors, which is a kind of full closed-loop control system. It should be noted that the six-degrees-of-freedom motion of more complicated mechanisms can also be measured by using more equally-spaced laser displacement sensors in combination. The precision tracking system is a 3-PRR planar parallel platform as shown in Figure 2a, which is composed of a static platform, mobile platform, sliding pair, revolving pair, and grating scale. The sliding pair is driven by an ultrasonic linear motor that can be used in a vacuum environment. The precision tracking system has three planar degrees of freedom, as shown in Figure 2a, and they are the *X*-axis translational freedom, *Y*-axis translational freedom, and rotational freedom around the *Z*-axis, which can be measured by three equally-spaced laser displacement sensors.

The 3-PRR planar parallel positioning platform includes a mechanical portion and an electric control portion. The mechanical portion is composed of three identical kinematic chains, with each chain including an ultrasonic linear motor, a connecting rod, a motor bracket, and a linear guide. The mobile platform is supported by three connecting rods of kinematic chains. The electric control portion consists of three laser displacement sensors, three linear grating encoders, photoelectric limit switch, servo drivers, computer, and motion card. Because the input of analogue signal is seriously influenced by external noise, the input of digital signal through the serial port was adopted. The type of the LDS was Keyence LK-H050, with a beam diameter of 50 μm, a measurement range of ±10 mm, and a sampling rate of 20 μs.

A schematic diagram of the 3-PRR parallel mechanism is shown in Figure 2b. The closed-loop vector method was used to analyse the kinematic chain. Because the sum of the closed loop vector is zero, the kinematic constraint relation can be expressed as:

(1) $$E_{i}F_{i}+F_{i}G_{i}+G_{i}M+MO+OE_{i}=0\;,\left(i=1,2,3\right)$$

where the locations of E, F, G, M, and O can be seen in Figure 2b; E is the fixed point, F is the first rotating pair, G is the second rotating pair, M is the geometric center of the mobile platform, and O is the geometric center of the static platform.

The projection along the *X*-axis and *Y*-axis of Equation (1) can be expressed by

(2) $$\left\{ \begin{array}{c} l_{i}\cos(\frac{2\pi (i-1)}{3})+h_{2}\cos\alpha_{i}+h_{3}\cos(\frac{\pi (4i-3)}{6}+\alpha_{M})=X_{M}-X_{Ei}\\ l_{i}\sin(\frac{2\pi (i-1)}{3})+h_{2}\sin\alpha_{i}+h_{3}\sin(\frac{\pi (4i-3)}{6}+\alpha_{M})=Y_{M}-Y_{Ei} \end{array}\right.$$

where $X_{Ei}$ and $Y_{Ei}$ are the coordinates of point $E_{i}$; $X_{M}$, $Y_{M}$, and $\alpha_{M}$ are the *X*-axis translational freedom, *Y*-axis translational freedom, and rotational freedom around the *Z*-axis, respectively; and $h_{1}$, $h_{2}$, and $h_{3}$ are the distance from point $E_{i}$ to point O, the length of the connecting rod, and the distance from point $G_{i}$ to point M, respectively. By solving Equation (2), the driving parameters $l_{i}$ and $\alpha_{i}$ and the driving input of every ultrasonic linear motor can be obtained for trajectory planning, which is known as inverse kinematics. For brevity, the expressions for *l_i_* and *α_i_* are omitted.

**Figure 3:**
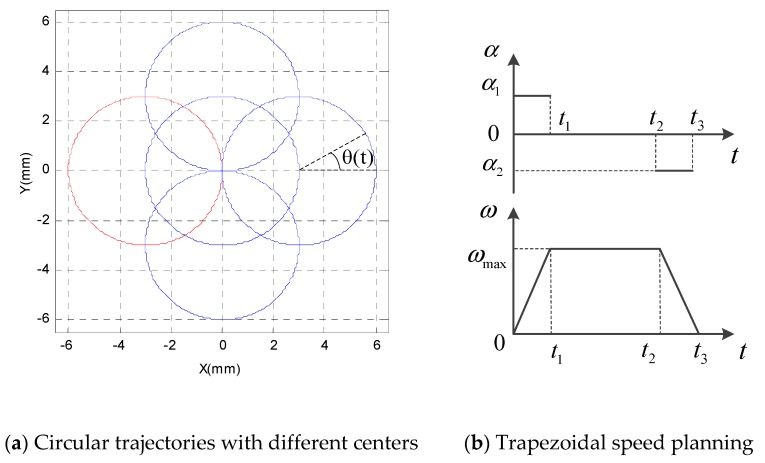
Different circular trajectories with radii of 3 mm.

To accurately locate the platform at the expected position, it is necessary to ensure that the trajectory motion of the parallel mechanism is smooth from the beginning to the end, and sudden changes of acceleration and deceleration should not happen. To reduce the impacts of vibration and shock, the position, speed, and acceleration of the parallel mechanism should be carefully designed. The trapezoidal speed planning algorithm was adopted in this paper, as shown in Figure 3b. The parallel platform was stationary at the starting position, then it accelerated to the maximum speed $\omega_{\max}$ at a given acceleration $\alpha_{1}$, ran at the maximum speed for a period of time, and finally slowed to the stop position at a given deceleration $\alpha_{2}$. During the whole process, the parallel platform had only a small amount of shock impact at times *t*_1_ and *t*_2_, which had limited influence on the tracking accuracy of the parallel mechanism. Values of *h*_1_ = 210.00 mm, *h*_2_ = 95.00 mm, and *h*_3_ = 28.00 mm were set to analyse the trajectory planning of the 3-PRR parallel mechanism. When trapezoidal speed planning was adopted, the mechanism was required to finish a circular motion in a time of 10 s with a constant attitude angle of *π*/3 rad, as shown in Figure 3a, where a circle with a radius of 3 mm and a center point of (−3, 0), denoted in red, is selected as an example to illustrate the control performances of different control algorithms. The acceleration time and deceleration time were set as:

(3) $$t_{1}=\frac{1}{4}t_{2}=\frac{1}{5}t_{3}=2\;s.$$

The angular velocity ω(t) of the moving platform of the 3-PRR planar parallel mechanism can be described by:

(4) $$\omega (t)=\{\begin{array}{l} \alpha_{1}*t,\;t\leq 2;\\ 2\alpha_{1},\;2<t\leq 8;\\ \alpha_{2}*(10-t),\;8<t\leq 10;\\ \alpha_{1}=\alpha_{2}=\pi /8; \end{array}$$

According to Equation (3), the angular displacement θ(t), position (x, y) and attitude angle φ of the moving platform can be expressed as:

(5) $$\theta (t)=\{\begin{array}{l} \alpha *t^{2}/2,\;t\leq 2;\\ \pi /4+2\alpha (t-2),\;2<t\leq 8;\\ 2\pi -\alpha {\left(10-t\right)}^{2}/2,\;8<t\leq 10; \end{array}$$

(6) $$\{\begin{array}{l} x=3\cos(\theta (t))-3;\\ y=3\sin(\theta (t));\\ \varphi =\pi /3; \end{array}$$

**Figure 4:**
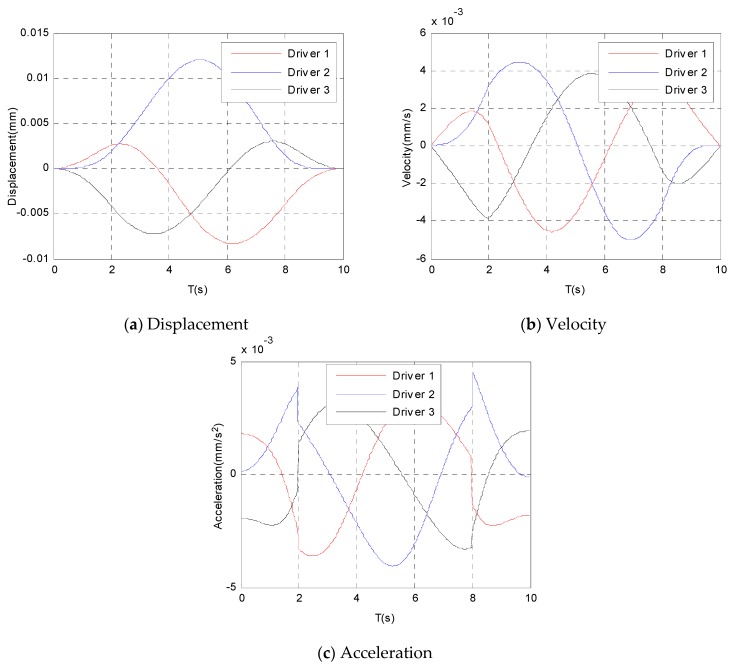
Displacement, velocity, and acceleration of the three motors.

By substituting Equations (4) and (5) into the equation of inverse kinematics, the time history of the displacement, velocity, and acceleration of the three driving motors can be obtained, which are shown in Figure 4 in detail.

From Figure 4a–c, it can be seen that all of the displacements and velocities change continuously and smoothly. However, the acceleration changes discontinuously, and little sudden acceleration changes occur at the times of 2 s and 8 s as shown in Figure 4c, from which one can see that the acceleration changes are not large enough to cause the mechanism vibration.

### 2.2. Deviation of Installing Angle and Equally-Spaced Sensors

**Figure 5:**
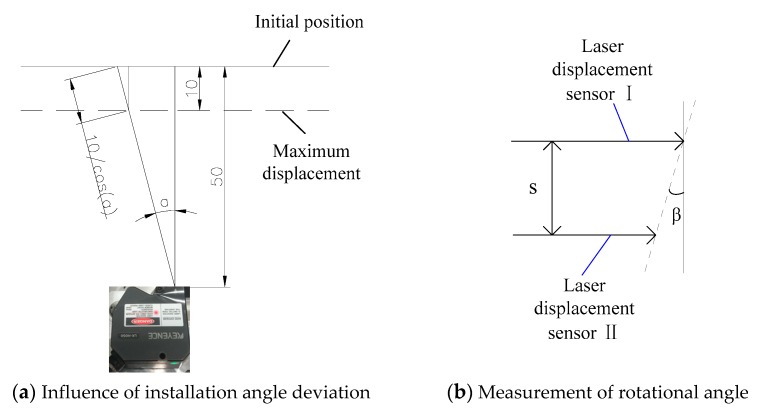
Influence of installation angle deviation on the measurement result and measurement of rotational angle.

Since measurement error has a large influence on the experimental result, it is important to avoid measurement error as much as possible. The beam of the laser displacement sensor should vertically strike the side of the measured block to ensure measurement accuracy. A standard block should then be used to position the laser perpendicular to the face of the measured block. A schematic diagram of the influence of deviation of the installing angle of the laser displacement sensor on the measurement data is shown in Figure 5a, from which the measurement error caused by the deviation of installing angle can be calculated by:

(7) Δl=l_real−l_ideal=10/cos(α)−10

where *α* is the deviation of installing angle, Δ*l* is the measuring error caused by deviation of installing angle, *l_real* is the actual measurement data, and *l_ideal* is the ideal measurement data. For example, when the deviations of installing angle are ±1° and ±2°, then the measuring errors Δ*l* can be calculated as $\Delta l=10/\cos(\pm 1^{{}^{\circ}})-10=0.0015$ mm and $\Delta l=10/\cos(\pm 2^{{}^{\circ}})-10=0.0061$ mm, respectively.

From the above analysis and Equation (6), one can see that when the value of deviation of installing angle is smaller than 1 degree, the measurement error is less than 0.0015 mm. However, when the value of installation angle deviation is larger than 2 degrees, the measurement error increases dramatically and seriously affects the measurement accuracy. Therefore, it is extremely important to limit and decrease the deviations of installing angles by using a standard block. Figure 5b shows the schematic diagram of the measurement of rotational angle. By placing two laser displacement sensors on the same side of the measured block, the rotational angle β of the 3-PRR parallel mechanism can be acquired by:

(8) $$\beta =\arctan[(l_{2}-l_{1})/s]$$

where $l_{1}$ and $l_{2}$ are the displacement values measured by laser displacement sensor I and laser displacement sensor II, respectively, and s is the distance between these two laser displacement sensors.

Let distance s be an independent variable and derive Equation (7) as:

(9) $$d(\arctan[(l_{2}-l_{1})/s])/ds=d(\arctan[\Delta l/s])/ds=-\Delta l/(s^{2}+\Delta l^{2})$$

There are three main factors influencing the distance s. First, it can be seen from the differential Equation (8) that, in order to improve the sensitivity of angle measurement, the distance s between the two laser heads should be set to be a small value appropriately. Second, because the laser displacement sensor is based on the principle of diffuse reflection of light, in order to prevent diffuse reflection from interfering with each other, the distance s should not be set too small. Third, to determine of the actual value of distance s, it is also necessary to consider the actual size of the measured block, as shown in Figure 1a with a length of 90 mm, which means that the value of distance s should be smaller than 90 mm. After considering all of these factors, it is appropriate to take the distance as 75 mm. It should be noted that the value of 75 mm is not an optimal value, but a reasonable value. Experiments show that when the value of distance s is set between 60 mm and 80 mm, one can achieve good measurement results. However, when the value of distance s is less than 60 mm, diffuse reflection can easily cause mutual interference.

### 2.3. Measurement Evaluation

**Figure 6:**
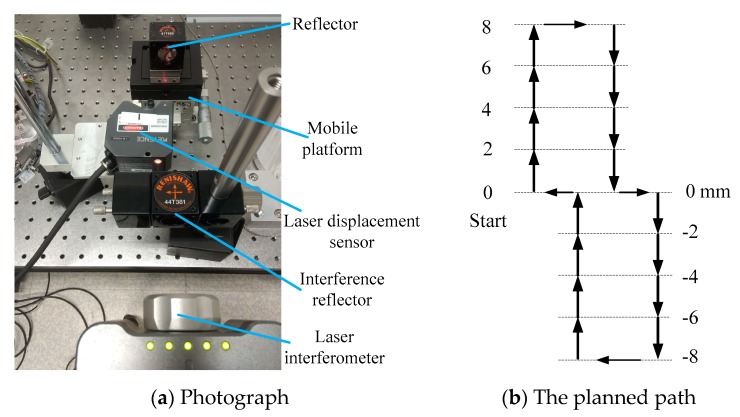
Photograph of the measurement comparison between the laser sensor and laser interferometer.

The above analysis shows that it is important to avoid measurement error as much as possible. To verify the measurement accuracy of the laser displacement sensor, a more precise measurement instrument should be adopted. The laser interferometer is a kind of high-accuracy measurement instrument with measurement accuracy reaching the nanometer level, which is more accurate than that of a laser displacement sensor. During the process of measurement evaluation, the interference reflector is fixed by magnetic adsorption to make both the interference reflector and the reflector locate on a same optical axis, as shown in Figure 6a. The laser interferometer and laser displacement sensor are both used to measure the movement of the parallel platform. To reduce the Abbe error and cosine error as much as possible, the laser interferometer should be carefully fixed.

Figure 6 presents the measurement comparison between the laser displacement sensor and laser interferometer. The planned path of the measurement evaluation is a line segment 16 mm long, as shown in Figure 6b. The starting point is located at the position of 0 mm, and the ending point is also located at the position of 0 mm after finishing the reciprocal motion. The process of the measurement evaluation is as follows. First, the master computer and motion card send commands to make the parallel platform continuously move to the positions of 0 mm, 2 mm, 4 mm, 6 mm, and 8 mm. The reciprocal motion then begins and continues until the mobile platform moves back to the position of 0 mm, as shown in Figure 6b. After finishing every step of 2 mm displacement, the measurement data of the laser displacement sensor and laser interferometer are recorded. At last, 16 points of location data are obtained after finishing the reciprocal motion, as shown in Table 1, which shows that the largest absolute values of the measurement difference between the laser displacement sensor and laser interferometer were smaller than 0.003 mm, except at the position of 4 mm. The large measurement difference at the position of 4 mm may have been caused by surface roughness and flatness, which can be largely eliminated by precision machining. In conclusion, after being fixed and regulated carefully, the laser displacement sensor can obtain high-accuracy measurements.

## 3. Feed-Forward Control

### 3.1. Correlation Analysis of Semi-Closed-Loop Tracking Errors

**Figure 7:**
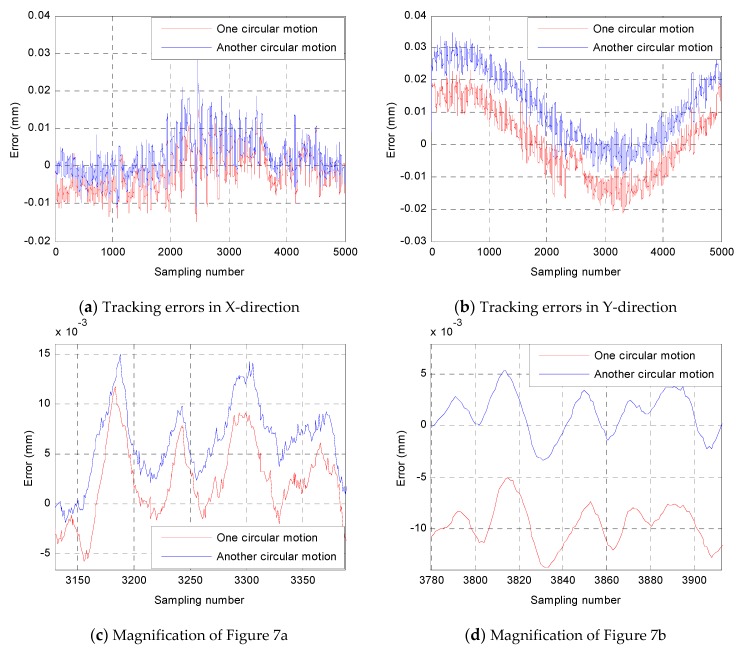
Semi-closed-loop tracking errors of two open-loop circular motions.

It is noteworthy that the experiments of feed-forward control, PI feedback control, and RBF neural network control were conducted on the basis of the full closed-loop control system having been built. Generally speaking, the realization of the measurement method is to lay the foundation for the control experiment. As we know, the feed-forward control is suitable for non-time-varying and stable systems. If a system is time-varying or greatly affected by external disturbances, the feed-forward control is not applicable to the system. Therefore, before conducting the experiment of feed-forward control, it was necessary to analyze the correlation of tracking errors of several semi-closed-loop control motions with the same trajectory. If there is a strong correlation between the tracking errors of several semi-closed-loop control motions, then it means that the system is basically a non-time-varying and stable system, and feed-forward control can be applied. In other words, the correlation analysis of semi-closed-loop errors is very meaningful, and can verify the feasibility of experimental application of feed-forward control as well as pointing out the main factors that influence the control effect. Correlation analysis of two semi-closed-loop circular motions is shown in Figure 7. A semi-closed-loop circular motion with a radius of 3 mm and a center of (0,3) is presented in this experiment. The semi-closed-loop errors can be expressed by:

(10) $$\{\begin{array}{c} e_{ix}(t)=x_{d}(t)-x_{i}(t)\\ e_{iy}(t)=y_{d}(t)-y_{i}(t) \end{array}$$

where $x_{d}(t)$ and $x_{i}(t)$ are the desired trajectory and actual trajectory in the X-direction respectively, and $e_{ix}(t)$ is the error in the X-direction. $y_{d}(t)$ and $y_{i}(t)$ are the desired trajectory and actual trajectory in the Y-direction respectively, and $e_{iy}(t)$ is the error in the Y-direction.

In order to analyze the correlation of semi-closed-loop errors clearly, the Pearson correlation coefficient is adopted to evaluate the correlation. Generally, the Pearson correlation coefficient $\rho_{x,y}$ can be expressed by:

(11) $$\rho_{x,y}=\frac{\mathrm{cov}(X,Y)}{\sigma_{x}\sigma_{y}}=\frac{E((X-\mu_{x})(Y-\mu_{y}))}{\sigma_{x}\sigma_{y}}=\frac{E(XY)-E(X)E(Y)}{\sqrt{E(X^{2})-E^{2}(X)}\sqrt{E(Y^{2})-E^{2}(Y)}}$$

Figure 7a,b present the semi-closed-loop tracking errors of two of the same circular motions in the *X* and *Y* directions, respectively. The Pearson correlation coefficients have been calculated to be 0.8764 and 0.9725 through Equation (10), and both of them have a kind of extremely strong positive correlation. Figure 7c,d present the magnification of Figure 7a,b respectively, from which one can see that the error-change trends of the two circular motions are almost the same. In conclusion, the correlation analysis has demonstrated that the semi-closed-loop errors of two same circular motions have a strong positive correlation, which also shows that the motion characteristics of the 3-PRR parallel platform are relatively stable.

### 3.2. Feed-Forward Control Experiment

**Figure 8:**
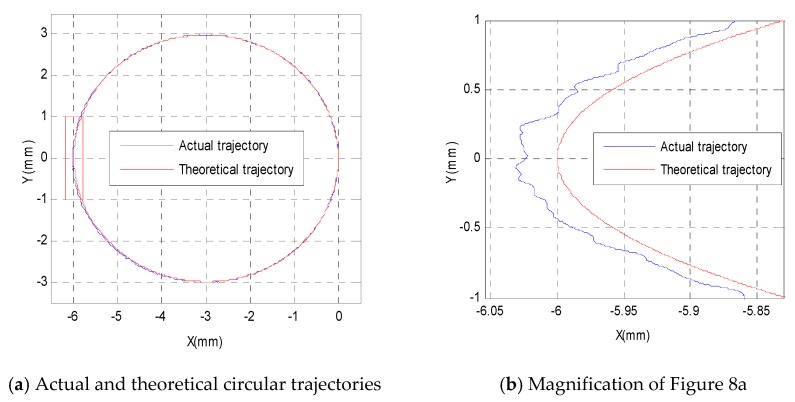

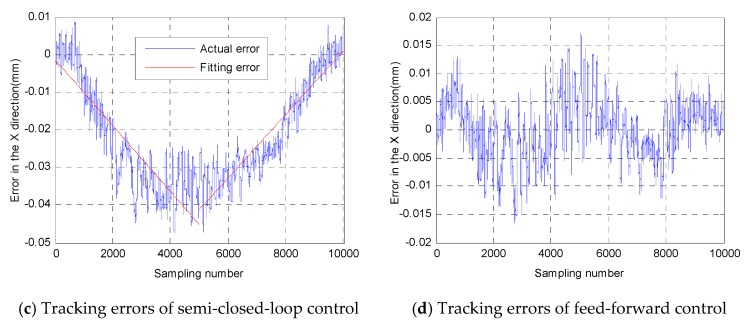
Semi-closed-loop control and feed-forward control results of a circular trajectory.

From the above correlation analysis of semi-closed-loop errors, one can see that the feed-forward control is suitable for the 3-PRR parallel platform. Figure 8a shows the semi-closed-loop control results of a circular trajectory with a radius of 3 mm, which includes the actual trajectory and theoretical trajectory. The red box in Figure 8a is magnified in Figure 8b to show the actual trajectory and theoretical trajectory in detail. The semi-closed-loop errors in the *X*-direction are shown in Figure 8c, which can be figured roughly by a fitting function marked in red. The semi-closed-loop errors in the *Y*-direction can also be figured roughly by a fitting function. In order to avoid repetitive explanation, only the fitting function description of *X*-direction errors was retained, and the feed-forward control was carried out. From Figure 8c, it can be seen that the fitting function can be selected as a linear function or quadratic function. After adopting an analysis of the least squares method, it is easy to see that the linear function is the best fitting function. The linear fitting function can then be shown as follows:

(12) $$\{\begin{array}{c} E_{x}=-0.00000877*S-0.00119,\;0\leq S<5000\\ E_{x}=0.00000846*S-0.08370,\;5000\leq S<10000 \end{array}$$

where $E_{x}$ is the error in the *X*-direction, $E_{y}$ is the error in the *Y*-direction, and S is the sampling number.

**Figure 9:**
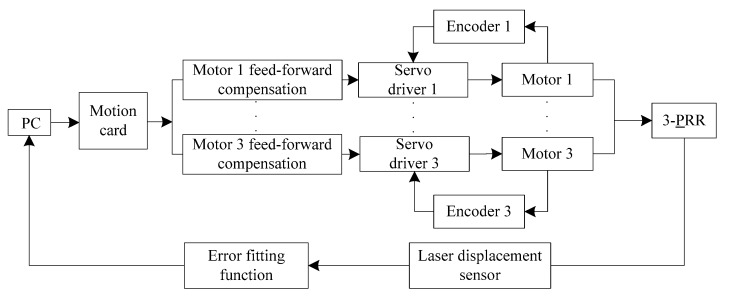
Schematic diagram of feed-forward control with the error fitting function.

From Equation (11), one can see that semi-closed-loop errors in the *X*-direction increase at 0.0017 mm every 200 sampling points when the sampling number is less than 5000, and the semi-closed-loop errors decrease at 0.0017 mm every 200 sampling points when the sampling number is greater than 5000. Therefore, before tracking the semi-closed-loop circular trajectory, the fitting error compensation should be inserted into the control input as feed-forward control, as shown in Figure 9; the semi-closed-loop errors in the *X*-direction will then be greatly reduced, as shown in Figure 8d. Figure 9 presents a schematic diagram of feed-forward control with the error fitting function, which is different from the closed-loop control method with the laser displacement sensor shown in Figure 1b. The feed-forward control results in the X-direction are shown in Figure 8d, which shows that the errors in the X-direction have been greatly reduced from a maximum of 0.0470 mm and a mean of 0.0220 mm to a maximum of 0.0170 mm and a mean of 0.0040 mm. Thus, the feed-forward control method can reduce semi-closed-loop errors to an extent. However, it is worth noting that when there is mechanical deformation, external disturbance, or shock, the tracking accuracy of the feed-forward control method will seriously deteriorate.

## 4. PI Feedback Control

**Figure 10:**
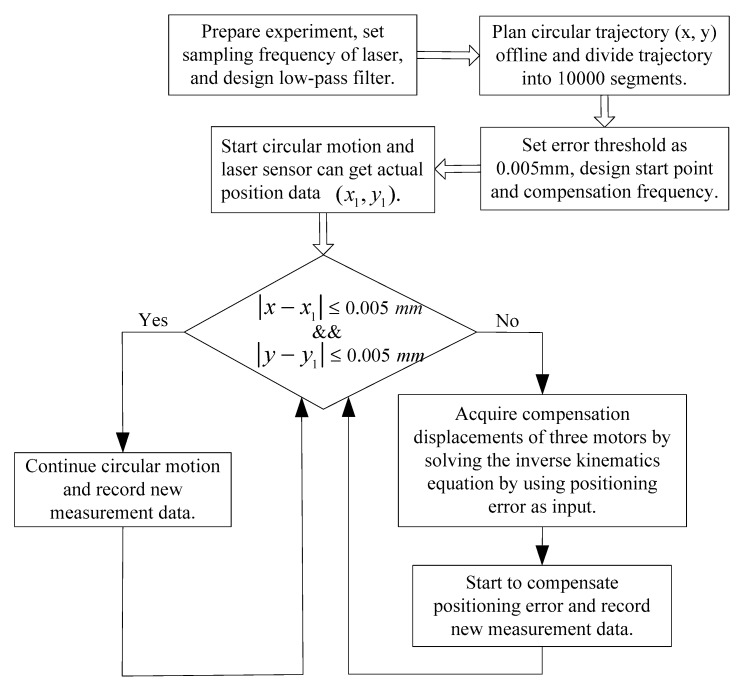
Flow chart of the closed-loop control experiment of the 3-PRR parallel platform.

The above feed-forward control experiment shows that feed-forward control can reduce tracking errors to an extent, but the control effect is not very satisfactory and the feed-forward control is strictly limited in range. On one hand, the error fitting function of feed-forward control is not very accurate, the system is not an absolutely non-time-varying system, and the system is affected by external disturbances. The tracking accuracy of feed-forward control experiment will then not be very high. On the other hand, the feedback control has no requirement for a non-time-varying system and absence of external disturbance, which means feedback control can achieve higher tracking accuracy than feed-forward control in the actual experiment. The main disadvantage of feedback control is that it will slow down the motion of the platform. Figure 10 presents the flow chart of the closed-loop control experiment of the 3-PRR parallel platform. The first step was to prepare the experiment, set the sampling frequency of the laser displacement sensor, and design a low-pass filter. The circular trajectory (*x*, *y*) was then planned offline and divided into 10,000 segments. Next, the error threshold was set as 0.005 mm, and the starting point and compensation frequency were designed. The circular motion was then started, and the laser displacement sensor could obtain actual position data $\left(x_{1},y_{1}\right)$. After the measurement, the tracking error values $\left|x-x_{1}\right|$ and $\left|y-y_{1}\right|$ should be used to compare with the error threshold of 0.005 mm. If both the tracking error values $\left|x-x_{1}\right|$ and $\left|y-y_{1}\right|$ are smaller than 0.005 mm, then the system will continue the circular motion and record new measurement data. However, if either of the tracking error values $\left|x-x_{1}\right|$ and $\left|y-y_{1}\right|$ are larger than 0.005 mm, then it is necessary to compensate for the tracking error. The compensation displacements of three motors can be acquired by solving the inverse kinematics equation using the tracking errors as input. After eliminating the tracking errors, the system can continue the circular motion and record new measurement data.

**Figure 11:**
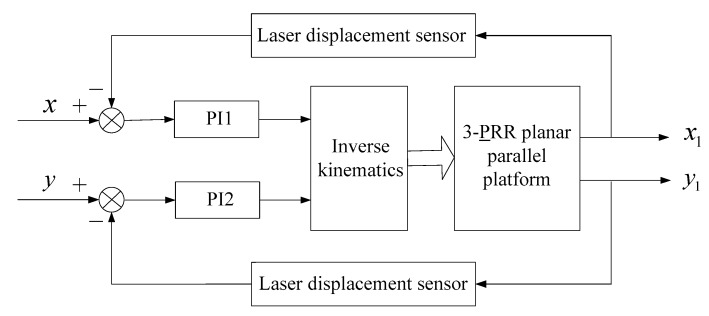
PI control of the 3-PRR parallel platform.

The main problem of the 3-PRR parallel platform is that this platform is a multi-input multi-output (MIMO) tracking system, which makes it difficult to establish an accurate mathematical model. For example, there are certain unknown influence factors that objectively exist, such as joint clearance, inertia force, and friction. To conduct the closed-loop control experiment with the laser displacement sensor, first, the conventional PI control algorithm was adopted and investigated as shown in Figure 11. Figure 11 presents the translations in the X-direction and Y-direction, which can be measured by two laser displacement sensors. The two controllers PI1 and PI2 were used to compensate the tracking error $x-x_{1}$ in the X-direction and tracking error $y-y_{1}$ in the Y-direction, respectively, which is typical of a multi-input multi-output control system.

**Figure 12:**
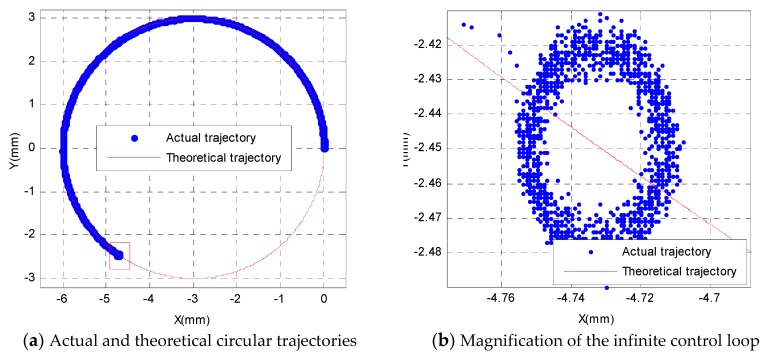
Circular trajectory tracking with a PI controller, $K_{p}=1,\;K_{i}=0$.

**Figure 13:**
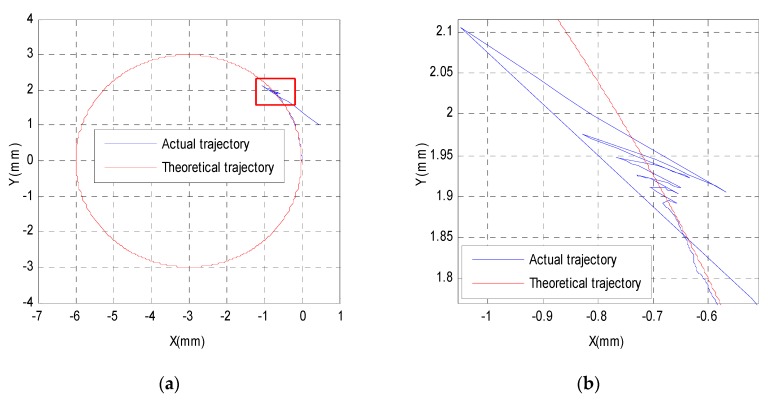
Circular trajectory tracking with a PI controller, $K_{p}=0.8,\;K_{i}=0$.

The experimental results of the PI control algorithm with $K_{p}=1,\;K_{i}=0$ are shown in Figure 12. From Figure 12a, one can see that the 3-PRR parallel platform can track the circular trajectory accurately at first, but the motion will enter an infinite loop, as shown in Figure 12b, which shows that the 3-PRR parallel platform cannot reach the error threshold after many repeats of the closed-loop control period, and the PI controller with $K_{p}=1,\;K_{i}=0$ cannot compensate every tracking error well when tracking the circular trajectory with a radius of 3 mm. In view of the above experimental result, other PI parameters can be selected to investigate the feedback control experiment, and the experimental result of a PI controller with $K_{p}=0.8,\;K_{i}=0$ is shown in Figure 13. From Figure 13a, one can see that the 3-PRR parallel platform can track the circular trajectory accurately at first, but the system soon loses control, as shown in Figure 13b, which also demonstrates that the 3-PRR parallel platform cannot track the circular trajectory accurately and the PI parameter settings of $K_{p}=0.8,\;K_{i}=0$ cannot compensate every tracking point well when tracking a circular trajectory with a radius of 3 mm.

**Figure 14:**
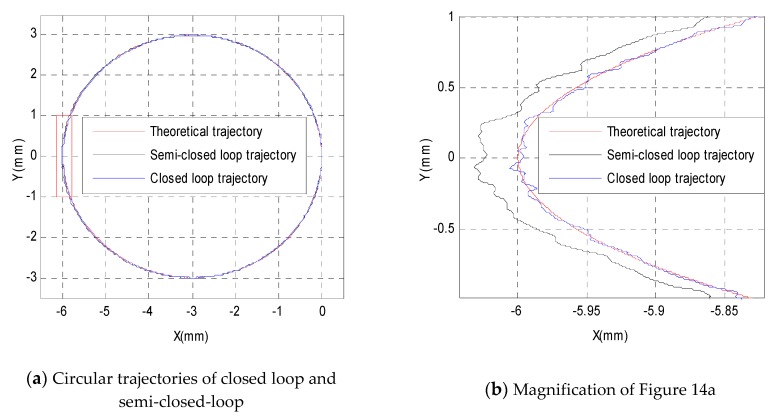

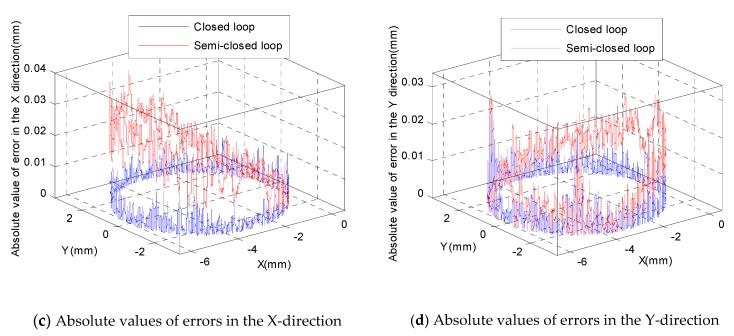
Circular trajectory tracking with a PI controller, $K_{p}=0.5,\;K_{i}=0.1$.

The above two experimental results show that the proportion term $K_{p}$ should be carefully set to be a relatively small value to avoid entering an infinite loop, as shown in Figure 12b, or losing control, as shown in Figure 13b. Meanwhile, if the proportion term $K_{p}$ alone cannot eliminate the steady-state error of the control system, then it is necessary to adopt the integration term $K_{i}$ to eliminate the steady-state error, compensate for the past deviation, and improve the stability of the control system. After several attempts, the PI parameters can be set to be $K_{p}=0.5,\;K_{i}=0.1$ to achieve a relatively good control effect, as shown in Figure 14, which shows the comparison between the circular trajectory tracking accuracies of different control methods, such as the semi-closed-loop control and closed-loop control. It should be noted that the parameters of $K_{p}=0.5,\;K_{i}=0.1$ are not optimal parameters, but reasonable parameters.

The theoretical trajectory, semi-closed-loop control trajectory, and closed-loop control trajectory are shown in Figure 14a. The red box in Figure 14a is magnified in Figure 14b, which shows the three trajectories in detail and is a kind of qualitative analysis. The quantitative analysis of the closed-loop control algorithm and semi-closed-loop control algorithm are presented in Figure 14c,d. The absolute values of tracking errors in the *X*-direction are shown in Figure 14c, and the absolute values of tracking errors in the *Y*-direction are shown in Figure 14d, which demonstrate that the absolute values of the error both in the *X*-direction and *Y*-direction were greatly reduced compared with the semi-closed-loop control algorithm. Compared with the tracking accuracy of the semi-closed-loop control, the tracking errors in the *X*-direction were greatly reduced from a maximum of 0.047 mm and a mean of 0.022 mm to a maximum of 0.016 mm and a mean of 0.0035 mm, respectively. The errors in the *Y*-direction were greatly reduced from a maximum of 0.035 mm and a mean of 0.012 mm to a maximum of 0.027 mm and a mean of 0.0042 mm, respectively. In conclusion, when the PI parameters are carefully selected to avoid entering an infinite loop or losing control, the conventional PI closed-loop control algorithm can achieve a relatively good control effect.

## 5. RBF Neural Network Control

### 5.1. RBF Neural Network

When the PI parameters are set to be $K_{p}=0.5,\;K_{i}=0.1$, a relatively good control effect can be achieved for a circular trajectory with a radius of 3 mm and a circular center of (−3,0), which is not a good enough control effect. In theory, different PI parameters should be used at different locations in order to achieve a better control effect, which was difficult to carry out in the experiment. In addition, there are many nonlinear factors involved in the 3-PRR parallel mechanism, such as load variation, mechanical deformation, and joint clearance, which necessitates a parameter tuning controller. RBF neural network control is based on PI feedback control. Most positions of tracking trajectory are controlled by constant PI parameters, and RBF neural network control is performed only at specific positions, such as the position in the red box in Figure 12b and Figure 13b. The position in the red box of Figure 12b and Figure 13b often corresponds to the position where the speed of any motor reaches its maximum value, as shown in Figure 4b. Therefore, RBF neural network control can achieve a higher tracking accuracy than PI feedback control. The disadvantage of RBF neural network control is that, in the first run, RBF neural network needs some time to learn online, which will reduce the speed of trajectory tracking. The advantage of RBF neural network is that, after the first run, it does not need any time to learn again to repeat the trajectory, and the first learning result can be used. An incremental PI controller was applied in the experiment and it can be expressed as:

(13) $$\{\begin{array}{l} u(k)=u(k-1)+\Delta u(k)\\ \Delta u(k)=K_{p}(e(k)-e(k-1))+K_{i}e(k) \end{array}$$

where Δu(k) is the incremental control of the PI controller, and e(k) is the tracking error.

**Figure 15:**
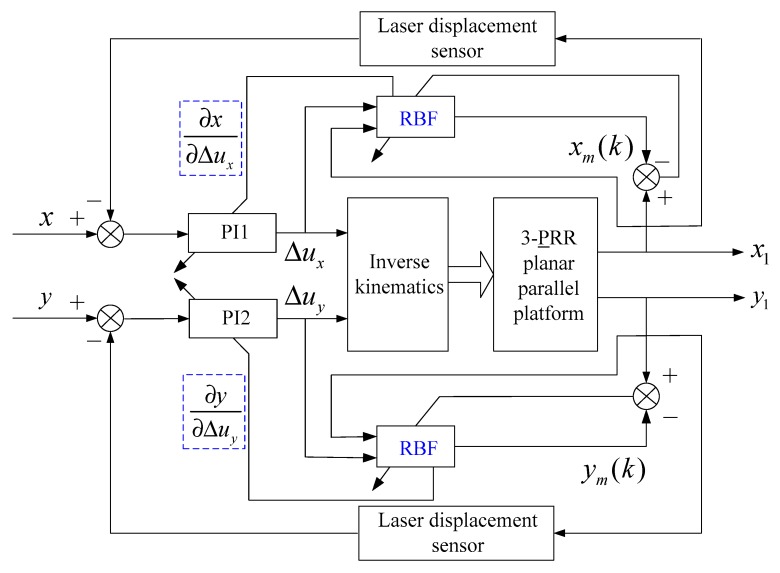
Radial basis function (RBF) neural network controller of the 3-PRR parallel platform.

**Figure 16:**
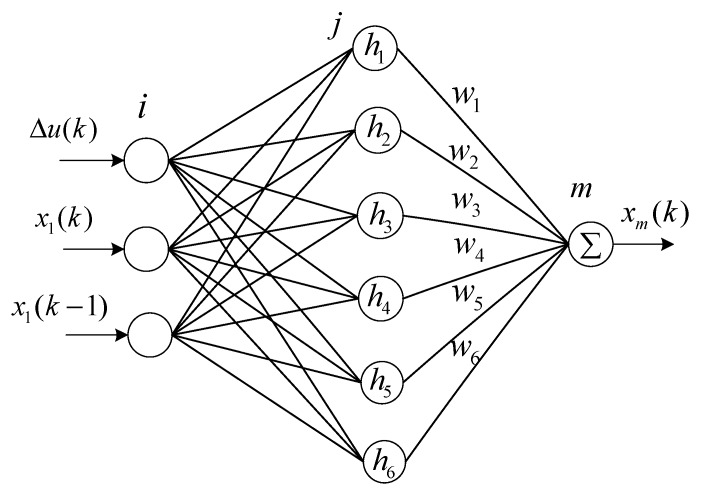
Structure of the three layers of the RBF neural network.

The RBF neural network controllers of the 3-PRR planar parallel platform are shown in Figure 15, which are marked in blue and designed to adjust the PI feedback controllers. Different from the BP neural network controller, which directly uses the output of the output layer as the PI parameters $K_{p}$ and $K_{i}$, the RBF neural network controller adopts the Jacobian function $\frac{\partial x(k)}{\partial \Delta u(k)}$ marked in the blue box to adjust the PI parameters $K_{p}$ and $K_{i}$, as shown in Figure 15. Since the RBF neural network controllers that adjust the PI parameters are almost the same, only the RBF neural network controller that compensates the tracking error $x-x_{1}$ in the X-direction was shown and investigated in this paper. Figure 16 presents the structure of the three layers of the RBF neural network, which includes input layer i with three neurons, hidden layer j with six neurons, and output layer m with one neuron. The control increment Δu(k) and measurement data $x_{1}(k)$ and $x_{1}(k-1)$ were used as the input of the input layer. The activation function $h_{j}$ of the hidden layer is the Gaussian radial basis function. The matrix $\left[w_{1},\;w_{2},\;w_{3},\;w_{4},\;w_{5},\;w_{6}\right]$ is the weight of the output layer, and $x_{m}(k)$ is the output of the RBF neural network. The PI parameters $K_{p}$ and $K_{i}$ were adjusted by the Jacobian function $\frac{\partial x(k)}{\partial \Delta u(k)}$ of the RBF neural network.

According to the gradient descent method, the adjustment of the weight, centric vector and basis breadth of the Gaussian radial basis function can be expressed by:

(14) $$\{\begin{array}{l} w_{j}(k)=w_{j}(k-1)+\eta (x_{1}(k)-x_{m}(k))h_{j}+\alpha (w_{j}(k-1)-w_{j}(k-2))\\ \Delta b_{j}=(x_{1}(k)-x_{m}(k))w_{j}h_{j}\frac{{\left\|X-C_{j}\right\|}^{2}}{{b_{j}}^{3}}\\ b_{j}(k)=b_{j}(k-1)+\eta \Delta b_{j}+\alpha (b_{j}(k-1)-b_{j}(k-2))\\ \Delta c_{ji}=(x_{1}(k)-x_{m}(k))w_{j}\frac{x_{j}-c_{ji}}{{b_{j}}^{2}}\\ c_{ji}(k)=c_{ji}(k-1)+\eta \Delta c_{ji}+\alpha (c_{ji}(k-1)-c_{ji}(k-2)) \end{array}$$

An incremental PI controller was applied in the experiment, and the adjustment of the PI parameters can be determined by:

(15) $$\{\begin{array}{l} E\left(k\right)=\frac{1}{2}{\left(e(k)\right)}^{2}=\frac{1}{2}{\left(x(k)-x_{1}(k)\right)}^{2}\\ \Delta K_{p}=-\eta \frac{\partial E}{\partial K_{p}}=-\eta \frac{\partial E}{\partial x}\frac{\partial x}{\partial \Delta u}\frac{\partial \Delta u}{\partial K_{p}}=\eta e(k)\frac{\partial x}{\partial \Delta u}(e(k)-e(k-1))\\ \Delta K_{i}=-\eta \frac{\partial E}{\partial K_{i}}=-\eta \frac{\partial E}{\partial x}\frac{\partial x}{\partial \Delta u}\frac{\partial \Delta u}{\partial K_{i}}=\eta e(k)\frac{\partial x}{\partial \Delta u}e(k) \end{array}$$

**Figure 17:**
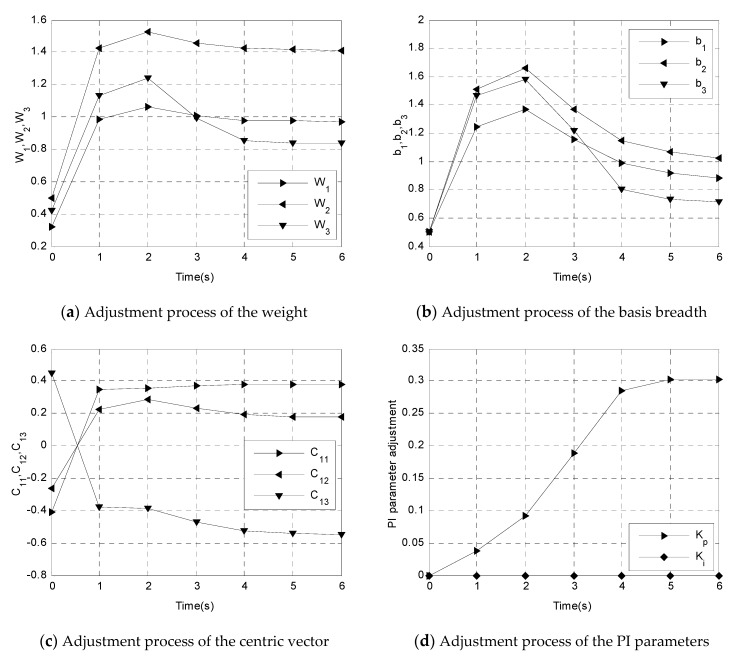
The adjustment process of the RBF neural network controller.

The definitions of certain given symbols are shown in Table 2. The adjustment process of the RBF neural-network controller is shown in Figure 17. To compensate for a large tracking error of the circular motion, the position of the red box in Figure 13a was selected for the experiment of RBF neura network control. The initial values of the output layer weight matrix were set to be [0.321, 0.499, 0.418, −0.075, 0.050, −0.418], which are random numbers between −0.5 and 0.5. The initial values of the basis breadth matrix of the Gaussian radial basis function were set to be [0.5, 0.5, 0.5, 0.5, 0.5, 0.5]. The initial values of the centric vector matrix of the Gaussian radial basis function were set to be [−0.412, −0.261, 0.447; −0.143, −0.089, 0.495; −0.294, −0.380, −0.413; 0.3874, −0.051, 0.027; −0.480, 0.305, 0.182; −0.465, −0.332, 0.375], which are random numbers between −0.5 and 0.5. The initial values of the PI parameters were set as $K_{p}=0,\;K_{i}=0$.

Figure 17a–c present a part of the adjustment process of the RBF neural network, which shows that the weights of the output layer and centric vector finished the adjustment process in 4 s. The values of the basis breadth converged relatively slowly and finished the adjustment process in 6 s. After the adjustment process was finished, the weight matrix of the output layer was [0.971, 1.412, 0.838, 1.160, 0.872, 0.240]. The values of the basis breadth matrix of the Gaussian radial basis function were [0.885, 1.026, 0.7104, 0.385, 0.623, 0.533]. The values of the centric vector matrix of the Gaussian radial basis function were [0.383, 0.176, −0.548; 0.290, 0.102, −1.156; 0.441, 0.183, 0.130; 0.144, −0.019, 0.010; 0.079, −0.048, −0.0304; −0.513, −0.366, 0.414]. Figure 17d shows that the PI parameters can finish the adjustment process in 4 s, which results in $K_{p}=0.302,\;K_{i}=0.01$ and demonstrates that the proportion term $K_{p}$ plays the main role when compensating for the tracking error.

### 5.2. Trajectory Tracking Experiment Based on RBF Neural Network

**Figure 18:**
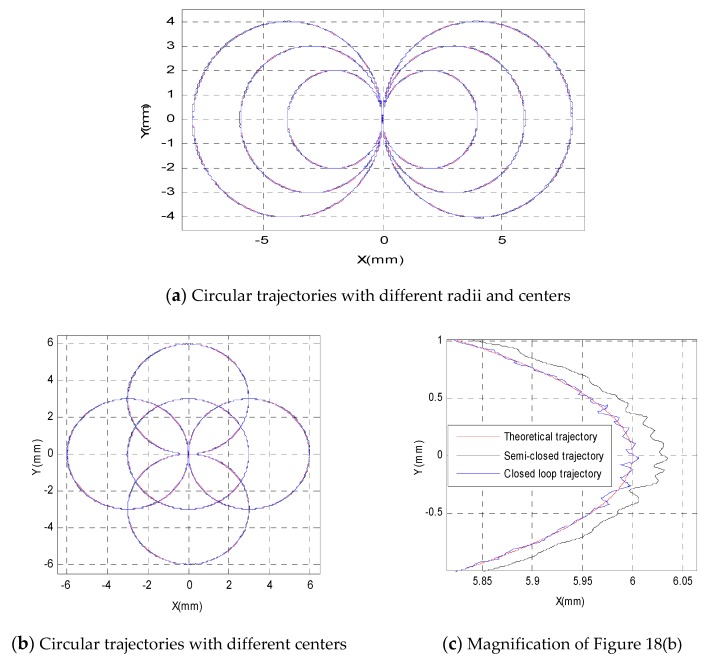

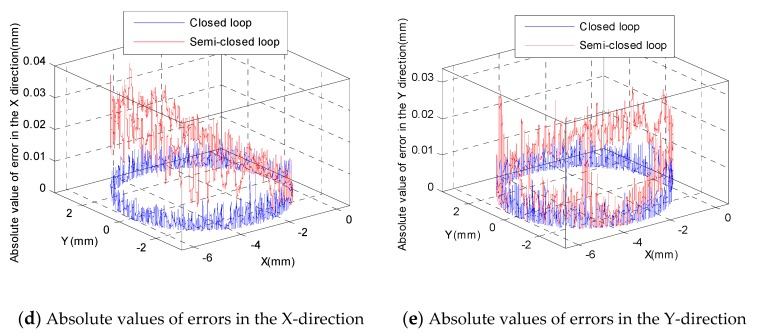
Neural-network-based experimental results of different circular trajectories.

After finishing the adjustment process of the RBF neural network, the trajectory tracking experiment based on RBF neural network was conducted. To verify the control performance of the RBF neural network controller when tracking different circular trajectories, the tracking experiments of different circular trajectories were conducted. The neural-network-based experimental results for tracking different circular trajectories are shown in Figure 18. Figure 18a presents the experimental results of several circular trajectories with different radii, such as radii of 2 mm, 3 mm, and 4 mm. Figure 18b presents the experimental results of tracking five circular trajectories with different circular centers, such as circular centers of (0, 0), (3, 0), (−3, 0), (0, 3), and (0, −3). The point at the position of 6 mm in the *X*-axis in Figure 18b is magnified in Figure 18c, which shows that the neural-network-based control algorithm can dramatically improve the tracking accuracy compared with the semi-closed-loop control algorithm. The qualitative comparison of the positioning precision is shown in Figure 18d,e, which demonstrates that the tracking accuracies both in the *X*-direction and *Y*-direction were improved by using the neural-network-based control algorithm.

A quantitative comparison of the tracking accuracies of the different control algorithms is presented in Table 3, which includes three evaluation indicators, namely, the mean value, standard deviation, and the maximum value of tracking errors. The circular trajectory with a radius of 3 mm and a center point of (−3,0) has been selected to illustrate the control performances of different control algorithms in Table 3. When the tracking accuracy of neural-network-based control is used to compare with that of the semi-closed-loop control, one can see that the errors in the *X*-direction are greatly reduced from a mean $X_{\mathrm{mean}}$ of 0.0220 mm and a maximum $X_{\max}$ of 0.0470 mm to a mean of 0.0025 mm and a maximum of 0.0177 mm. The errors in the Y-direction are greatly reduced from a mean $Y_{\mathrm{mean}}$ of 0.0120 mm and a maximum $Y_{\max}$ of 0.0350 mm to a mean of 0.0026 mm and a maximum of 0.0206 mm. When the tracking accuracy of neural network-based control is used to compare with that of PI feedback control with $K_{p}=0.5,\;K_{i}=0.1$, the mean errors in the X-direction and Y-direction are reduced from 0.0032 mm and 0.0037 mm to 0.0025 mm and 0.0026 mm, respectively. It should be noted that the PI parameters $K_{p}=0.5,\;K_{i}=0.1$ are carefully selected by many attempts to avoid entering an infinite loop as shown in Figure 12b or losing control as shown in Figure 13b. In conclusion, the experimental results demonstrate that all of the feed-forward control, PI feedback control, and RBF neural-network control algorithms have better control effects than that of semi-closed-loop control, which proves the validity of the designed full closed-loop control system based on a combination of laser displacement sensors and linear grating encoders. In addition, the experimental results also demonstrate that the control effect of feed-forward control is better than that of semi-closed-loop control; the control effect of PI feedback control is better than that of feed-forward control; and the control effect of RBF neural-network control is better than that of PI feedback control. In addition, in the first run, the RBF neural network control took more time than the PI feedback control. After the first run, the RBF neural-network control took the same time as the PI feedback control. The tracking times of different control algorithms are shown in Table 4. Because there was no feedback control, the semi-closed-loop control and feed-forward control took relatively less time.

## 6. Conclusions

A three-degrees-of-freedom full closed-loop control precision tracking system was developed and analyzed in this paper. Equally-spaced laser displacement sensors and linear grating encoders were used in combination not only for measurement but also for feedback control. First, the kinematics model of the 3-PRR parallel mechanism was analyzed, which showed that a constraint relation between the output value and input value can be established by solving the inverse kinematics equation. The precision tracking system with three equally-spaced laser displacement sensors and linear grating encoders for full closed-loop control was then introduced. Before conducting the closed-loop feedback control experiment, a feed-forward control method was applied. After that, several experiments using the PI feedback control algorithm were conducted. To improve the absolute tracking accuracy of the closed-loop control system, a RBF neural network control algorithm was designed and applied. Finally, the experimental results demonstrate that the control effect of feed-forward control is better than that of semi-closed-loop control; the control effect of PI feedback control is better than that of feed-forward control; and the control effect of RBF neural network control is better than that of PI feedback control. It should be noted that other experiments have been done to run rhombus trajectories with sharp edges, which have shown similar control effects to circular trajectories. In order to ensure the concision of this article, the experiments of rhombus trajectories have been omitted.

## Figures and Tables

**Table 1:** Measurement evaluation between the laser sensor and laser interferometer.

Evaluation Experiment	Ideal Input Displacement (mm)	Laser Sensor Measurement *i*	Laser Interferometer Measurement *j*	|i−j|
1	2.000	2.016	2.014	0.002
2	4.000	4.030	4.022	0.008
3	6.000	6.037	6.035	0.002
4	8.000	8.041	8.044	0.003
5	6.000	6.037	6.035	0.002
6	4.000	4.029	4.022	0.007
7	2.000	2.014	2.014	0.000
8	0.000	0.002	0.000	0.002
9	−2.000	−2.015	−2.014	0.001
10	−4.000	−4.027	−4.027	0.000
11	−6.000	−6.039	−6.039	0.000
12	−8.000	−8.053	−8.054	0.001
13	−6.000	−6.039	−6.039	0.000
14	−4.000	−4.029	−4.028	0.001
15	−2.000	−2.017	−2.015	0.002
16	0.000	−0.004	−0.002	0.002

**Table 2:** Definitions of the given symbols of the RBF neural network.

Symbol	Meaning
$h_{i}(x)$	Gaussian radial basis function
$C_{j}$	Centric vector of the Gaussian radial basis function
$b_{j}$	Basis breadth of the Gaussian radial basis function
$w_{j}$	Weight of the output layer
$x_{m}(k)$	Output of the RBF neural network
α	Inertia coefficient
η	Learning rate
E(k)	Performance index function
$\frac{\partial x(k)}{\partial \Delta u(k)}$	Jacobian function used to adjust the PI parameter

**Table 3:** Tracking accuracies of different control algorithms.

Control Algorithm	$X_{\mathrm{mean}}\;(\mathrm{mm})$	$X_{std}\;(\mathrm{mm})$	$X_{\max}\;(\mathrm{mm})$	$Y_{\mathrm{mean}}\;(\mathrm{mm})$	$Y_{std}\;(\mathrm{mm})$	$Y_{\max}\;(\mathrm{mm})$
Semi-closed-loop control	0.0220	0.0170	0.0470	0.0120	0.0090	0.0350
Feed-forward control	0.0040	0.0033	0.0170	0.0045	0.0038	0.0210
PI control with $K_{p}=0.5,\;K_{i}=0.1$	0.0032	0.0026	0.0160	0.0037	0.0031	0.0274
RBF neural network control	0.0025	0.0020	0.0177	0.0026	0.0020	0.0206

**Table 4:** Tracking time of different control algorithms.

Control Algorithm	Time of First Run (s)	Time of Later Run (s)
Semi-closed-loop control	10	10
Feed-forward control	10	10
PI control with $K_{p}=0.5,\;K_{i}=0.1$	20	20
RBF neural network control	36	20
